# Carbon monoxide is involved in melatonin-enhanced drought resistance in tomato seedlings by enhancing chlorophyll synthesis pathway

**DOI:** 10.1186/s12870-024-04793-3

**Published:** 2024-02-09

**Authors:** Yunzhi Liu, Junrong Xu, Xuefang Lu, Mengxiao Huang, Yuanzhi Mao, Chuanghao Li, Wenjin Yu, Changxia Li

**Affiliations:** College of Agriculture, Guangxi University, Nanning, 530004 China

**Keywords:** Carbon monoxide, Melatonin, Drought stress, Chlorophyll synthesis, Genes expression

## Abstract

**Background:**

Drought is thought to be a major abiotic stress that dramatically limits tomato growth and production. As signal molecule, melatonin (MT) and carbon monoxide (CO) can enhance plant stress resistance. However, the effect and underlying mechanism of CO involving MT-mediated drought resistance in seedling growth remains unknown. In this study, tomato (*Solanum lycopersicum* L. ‘Micro-Tom’) seedlings were used to investigate the interaction and mechanism of MT and CO in response to drought stress.

**Results:**

The growth of tomato seedlings was inhibited significantly under drought stress. Exogenous MT or CO mitigated the drought-induced impairment in a dose-dependent manner, with the greatest efficiency provided by 100 and 500 µM, respectively. But application of hemoglobin (Hb, a CO scavenger) restrained the positive effects of MT on the growth of tomato seedlings under drought stress. MT and CO treatment promoted chlorophyll a (Chl a) and chlorophyll a (Chl b) accumulations. Under drought stress, the intermediate products of chlorophyll biosynthesis such as protoporphyrin IX (Proto IX), Mg-protoporphyrin IX (Mg-Proto IX), potochlorophyllide (Pchlide) and heme were increased by MT or CO, but uroporphyrinogen III (Uro III) content decreased in MT-treated or CO-treated tomato seedlings. Meanwhile, MT or CO up-regulated the expression of chlorophyll and heme synthetic-related genes *SlUROD*, *SlPPOX*, *SlMGMT*, *SlFECH*, *SlPOR*, *SlChlS*, and *SlCAO*. However, the effects of MT on chlorophyll biosynthesis were almost reversed by Hb.

**Conclusion:**

The results suggested that MT and CO can alleviate drought stress and facilitate the synthesis of Chl and heme in tomato seedlings. CO played an essential role in MT-enhanced drought resistance via facilitating chlorophyll biosynthesis pathway.

## Background

Drought is a widely-present threat to the plant growth, development, and production, which limits agricultural productivity, geographical distribution and survival of plant [1]. Photosynthesis plays a vital role in plant physiology, which can be severely affected by drought [2]. Drought stress induces a decrease in photosynthesis-related parameters including photosynthetic pigments, stomatal conductance, intercellular CO_2_ concentration, and photosynthetic electron transfer rate in plant [2–4]. Additionally, drought can also trigger excessive production of reactive oxygen species (ROS) in plant cells, resulting in cellular membranes damage, electron leakage, and lipid peroxidation [5, 6]. In order to resist the drought stress, exploring efficient and eco-friendly growth regulators and their regulatory mechanisms is highly necessary.

Melatonin (MT), known as *N*-acetyl-5-methoxytryptamine, is a natural and pleiotropic indoleamine molecule and exists widely in various plant species and tissues [7]. In higher plant, MT acts as a positive regulator in seed germination [8], root growth [9], plant development [10], fruit ripening [11], postharvest storage [12], quality maintenance [13], and leaf senescence [14]. In addition, MT participates in multiple physiological and metabolic processes to enhance the resistance to abiotic stress, such as heat [15], alkaline [16], chilling [17], cadmium [18], and salt [19]. Under drought stress, seed priming with MT enhanced germination rate, root length, shoot length, root-shoot length ratio, and fresh seedling weight in maize [20]. MT could also alleviate drought stress by promoting water conservation, reducing electrolyte leakage, suppressing abscisic acid (ABA) synthesis, and maintaining cellular redox homeostasis in apple [21–23]. Meanwhile, MT neutralizes the inhibition of photosynthesis caused by drought stress [10, 24, 25]. Exogenous MT improved photosynthesis via increasing water holding capacity, chlorophyll content, net photosynthesis rate, transpiration rate, and stomatal conductance, facilitating electron transport in photosystem II (PS II) and gas exchange, maintaining cell turgor and intact grana lamella of chloroplast when plant was exposed to drought stress [4, 10, 26, 27]. Thus, exogenous application of MT is often implicated as a means of ameliorating the detrimental effects of drought stress.

Carbon monoxide (CO), an important signal and gaseous molecule, performs a variety of physiological functions in plant [28]. For example, application of exogenous CO positively regulates seed germination [29], lateral root formation [30], adventitious root development [31], root hair development [32], root elongation [33], and stomatal closure [34]. CO can be induced by different stresses, such as osmosis [35], salt [36], cadmium [37], mercury [38], ultraviolet-B radiation [39] and iron deficiency [40]. Moreover, Liu et al. [35] reported that CO enhanced the activities of antioxidant enzymes including superoxide dismutase (SOD), catalase (CAT), ascorbate peroxidase (APX), and dehydroascorbate reductase (DHAR) under drought stress in wheat. Exogenous CO improved photosynthetic capacity by maintaining chlorophyll content, elevating photochemical efficiency of photosystem II (PS II), PS II actual photochemical efficiency, and photochemical quench coefficient in cucumber under drought stress [41].

MT and CO are signal molecules, which involve in plant response to abiotic stress, generally requiring the involvement of other signal molecules or plant hormones [42, 43]. The density and elongation of tomato root hair were promoted by CO, due to the crosstalk with auxin, ethylene (ETH) and nitric oxide (NO) [32]. She and Song [34] demonstrated that hydrogen peroxide (H_2_O_2_) participated in CO-induced stomatal closure. CO was involved in H_2_-induced adventitious rooting under drought stress [41]. The chilling and salt tolerances can be enhanced by CO due to the improvement of NO-mediated redox homeostasis and ion homeostasis [44, 45]. In addition, MT treatment significantly increased the tolerance of drought via suppressing abscisic acid (ABA) synthesis and scavenging H_2_O_2_ [22]. Under salt stress, MT maintained the K^+^/Na^+^ homeostasis of seedling roots through H_2_S signal [46]. Exogenous MT promoted NO synthesis under heat stress, thus activating ascorbate-glutathione cycle [47]. Besides, NO was identified as a downstream signal molecule in MT-enhanced tolerance of alkaline stress [48].

Chlorophyll is a unique tetrapyrrole in higher plant, and performs an essential function in photosynthesis. Notably, chlorophyll and heme share a multistep, enzymatic biosynthetic pathway [49]. Uroporphyrinogen III (Uro III) is the first closed macrocycle in the pathway and the last common precursor of all tetrapyrroles [49, 50]. Under the catalysis of uroporphyrinogen decarboxylase (UROD), coproporhyrinogen III oxidase (COPX), and protoporphyrinogen IX oxidase (PPOX), Uro III is gradually converted to protoporphyrin IX (Proto IX). There are two major pathways for Proto IX transformation: synthesis of heme catalyzed by Fe-chelatase (FECH) or synthesis of Mg-protoporphyrin IX (Mg-Proto IX) catalyzed by Mg-chelatase (MGCH). In the Chl synthetic branch, Mg-Proto IX can be further catalyzed to form Mg-protoporphyrin IX monomethyl ester (MgPME) by Mg-protoporphyrin IX methyltransferase (MGMT). In the next step, MgPME is converted to protochlorophyllide (Pchlide), then protochlorophyllide oxidoreductase (POR) and chlorophyll synthase (ChlS) catalyze Pchlide to form chlorophyllide (Chlide) and chlorophyll a (Chl a) successively. Finally, Chl a can transform into chlorophyll b (Chl b) by chlorophyll a oxygenase (CAO) [51]. Drought or osmotic stress not only decrease chlorophyll content, but also decrease some crucial biosynthetic precursor molecules in porphyrin or chlorophyll metabolism including 5-aminolevulinic acid (5-ALA), porphobilinogen (PBG), Proto IX, Mg-Proto IX, and Pchlide in Kentucky bluegrass seedlings [52]. Analogously, in the study of Dalal et al. [53], chlorophyll biosynthesis was significantly down-regulated due to water stress during rice seedling development. Liu et al. [2] demonstrated that the expression of genes related to chlorophyll biosynthesis and photosynthesis including *Aradu.G22I6*, *Aradu.53,538*, *Aradu.ZV73M*, *Aradu.Z9Z80*, and *Aradu.LW197* were down-regulated in peanut under drought stress. As mentioned above, both MT and CO applications improve plant growth and photosynthesis under stress [14, 41]. Although previous studies about the mechanisms for MT and CO alleviating abiotic stress have focused on antioxidant capacity, ROS levels, photosynthetic rate, stomatal behavior, chlorophyll levels, fluorescence parameters, and etc., less attention being paid to the effects of MT and CO on chlorophyll biosynthetic pathway [15, 22, 41]. Additionally, the signal role of MT and CO in enhancing drought resistance during seedling growth is still poorly understood. Therefore, the objective of our study is to investigate the effects of MT and CO on the growth and chlorophyll biosynthesis in tomato seedlings under drought stress, as well as the relationship between MT and CO during this process.

## Results

### Tomato seedlings response to different degrees of drought stress

**Fig. 1:**
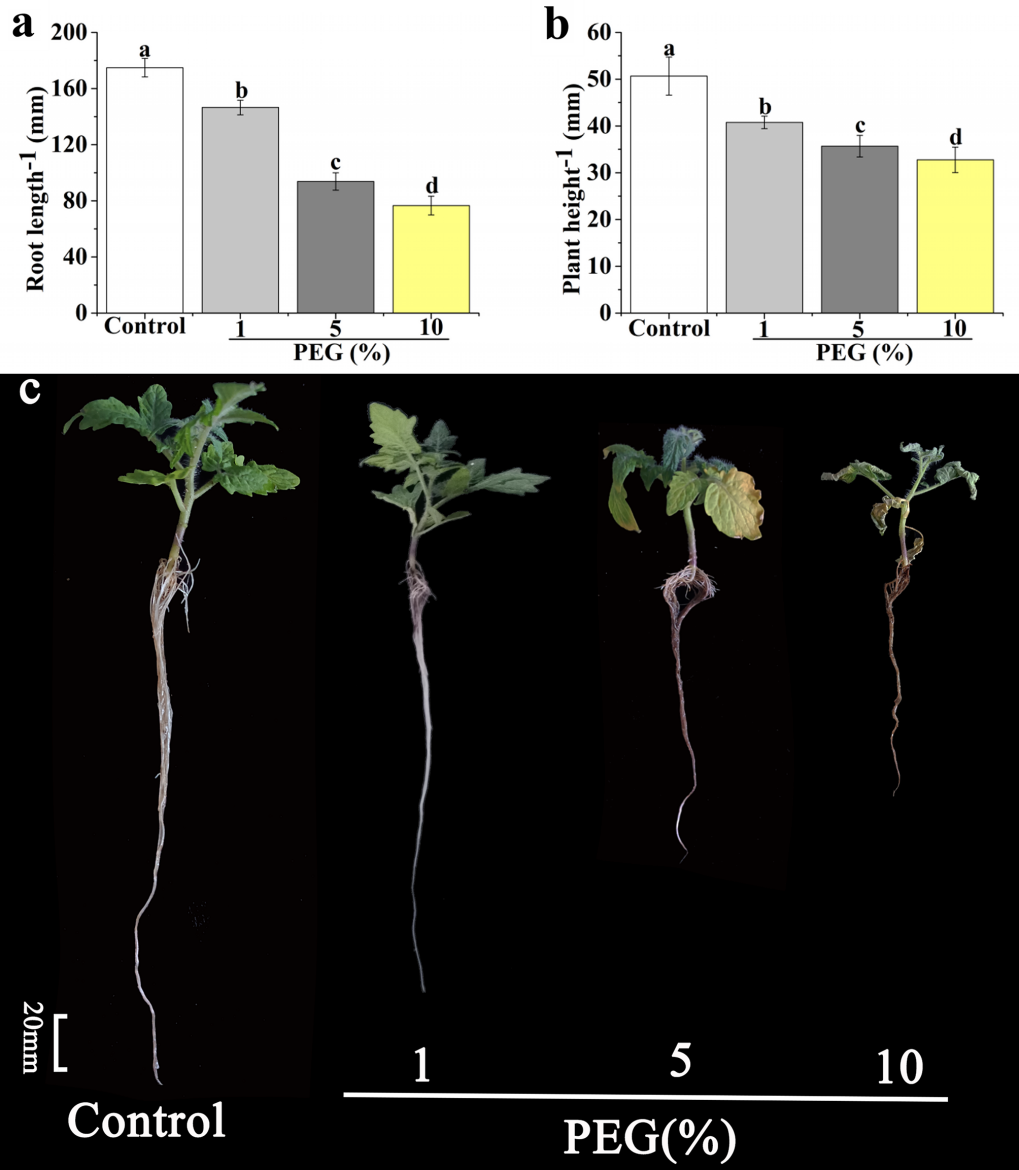
Effects of different concentrations of PEG (0, 1%, 5%, and 10%) on root length **(a)** and plant height **(b)** of tomato seedlings. Photographs **(c)** showed the tomato seedlings were incubated in the Hoagland solution containing different concentrations of PEG for 7 days. The data are the average of three replicates and are presented as means ± SE. Different letters indicate significant differences at *P* < 0.05 according to Duncan’s multiple range tests

Under drought stress, the root length of tomato seedlings was decreased significantly compared with the control (Fig. 1a and c). Root length of 1%, 5%, and 10% PEG treatments was decreased by 17.14%, 45.71%, and 56.11%, respectively, compared with the control. As shown in Fig. 1b and c, plant height of tomato seedlings suffering from drought stress decreased significantly compared with the control. Plant height of 1%, 5%, and 10% PEG treatments was decreased by 19.23%, 30.77%, and 38.46%, respectively, compared with the control. Therefore, treatments with 1%, 5%, and 10% PEG could be regarded as mild, medium, and severe drought stress, respectively. The current study used the 5% PEG to simulate medium drought stress in the subsequent experiment.

### Effects of different MT concentrations on the growth of tomato seedlings under drought stress

**Fig. 2:**
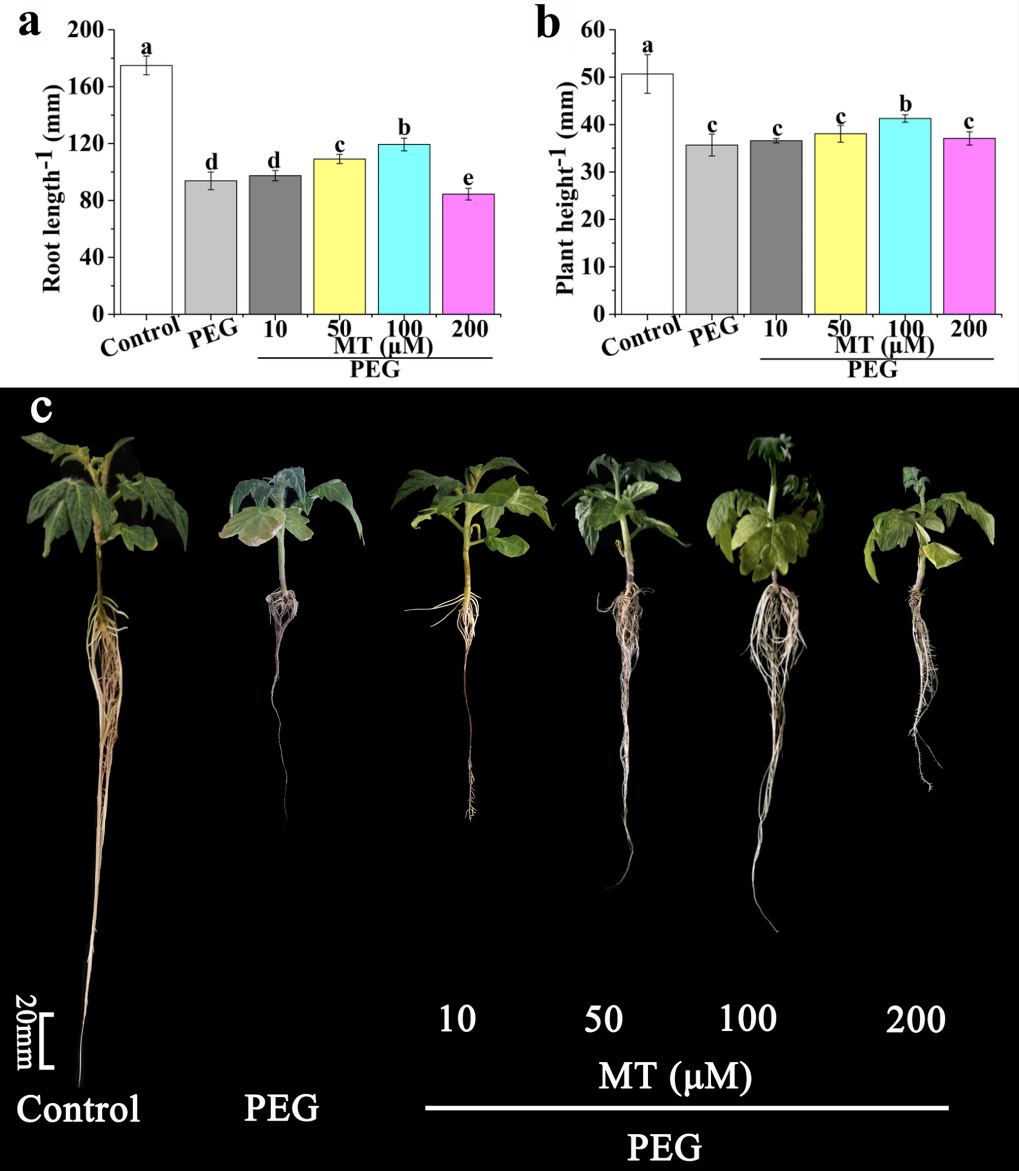
Effects of different concentrations of MT (0, 10, 50, 100, and 200 µM) on root length **(a)** and plant height **(b)** of tomato seedlings under drought stress. Photographs **(c)** showed the tomato seedlings were incubated in the mixture of Hoagland solution and 5% PEG containing different concentrations of MT for 7 days. The data are the average of three replicates and are presented as means ± SE. Different letters indicate significant differences at *P* < 0.05 according to Duncan’s multiple range tests

As shown in Fig. 2a and c, there was no significant difference in root length between PEG + 10 µM MT and PEG treatment. Application of 50 and 100 µM MT significantly improved root length by 14.58% and 25.11%, respectively, compared with PEG treatment (Fig. 2a). However, root length of PEG + 200 µM MT treatment was decreased by 12.50%, compared with PEG treatment. Moreover, only 100 µM MT treatment significantly increased plant height under drought stress, and the percentage of increase was 16.67% (Fig. 2b and c). Thus, 100 µM MT treatment was selected in the following experiment.

### Effects of different CO concentrations (supplied by hemin) on the growth of tomato seedlings under drought stress

**Fig. 3:**
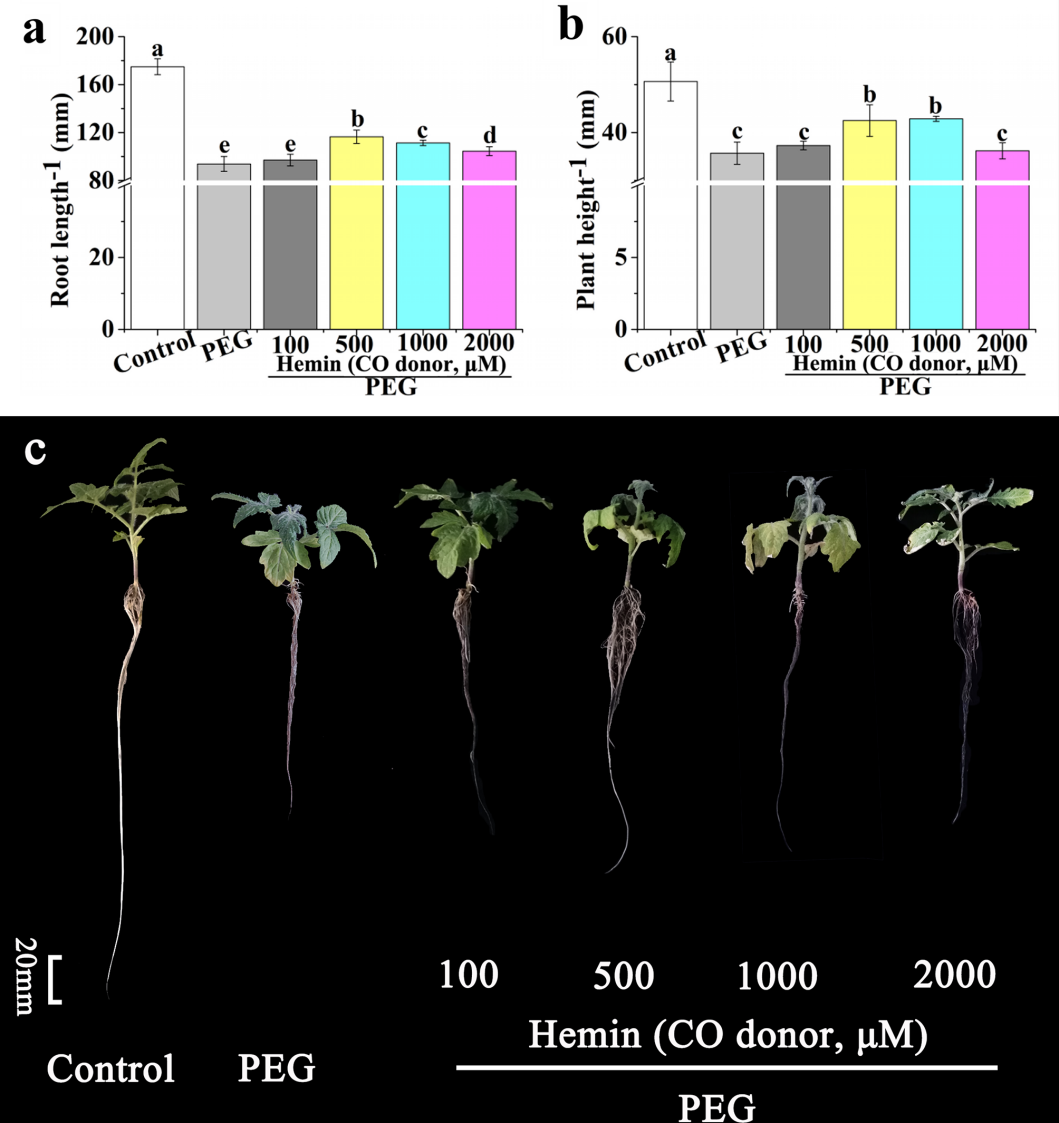
Effects of different concentrations of hemin (0, 100, 500, 1000, and 2000 µM) on root length **(a)** and plant height **(b)** of tomato seedlings under drought stress. Photographs **(c)** showed the tomato seedlings were incubated in the mixture of Hoagland solution and 5% PEG containing different concentrations of hemin for 7 days. The data are the average of three replicates and are presented as means ± SE. Different letters indicate significant differences at *P* < 0.05 according to Duncan’s multiple range tests

As shown in Fig. 3a, there was no significant difference in root length between PEG + 100 µM hemin and PEG treatment. As compared with PEG treatment, root length of PEG + 500 µM hemin, PEG + 1000 µM hemin, and PEG + 2000 µM hemin treatments were significantly increased by 23.96%, 17.71%, and 10.41%, respectively (Fig. 3a and c). Plant height of PEG + 500 µM hemin and PEG + 1000 µM hemin treatments were significantly increased by 16.67% and 19.44%, respectively, compared with PEG treatment (Fig. 3b). Among the various concentrations of hemin, 500 µM hemin treatment which showed the most positive effect on alleviating drought stress was regarded as CO treatment in further studies.

### Correlation between MT and CO in drought resistance of tomato seedlings

**Fig. 4:**
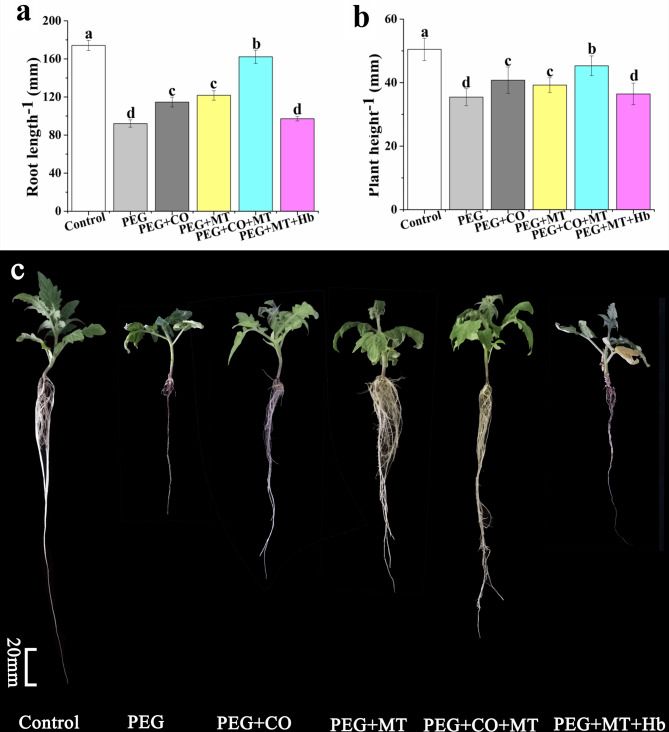
Effects of MT, CO, MT + CO, and MT + Hb on root length **(a)** and plant height **(b)** of tomato seedlings under drought stress. Photographs **(c)** showed the tomato seedlings treated by 7 days. The data are the average of three replicates and are presented as means ± SE. Different letters indicate significant differences at *P* < 0.05 according to Duncan’s multiple range tests

As shown in Fig. 4, the root length and plant height were remarkably decreased under drought stress. CO or MT treatment significantly increased root length under drought stress, and the percentages of increase were 25.53% and 27.66%, respectively (Fig. 4a and c). Compared with CO or MT alone treatment, CO and MT co-treatment significantly increased root length by 36.44% and 34.17%, respectively. To understand the relationship between CO and MT in drought resistance of tomato seedlings, the CO scavenger hemoglobin (Hb) was applied. The root length of PEG + MT + Hb treatment was decreased by 20.12% compared with PEG + MT treatment. Moreover, CO or MT treatment significantly increased plant height under drought stress, and the percentages of increase were 16.67% and 11.11%, respectively. Compared with CO or MT alone treatment, CO and MT co-treatment significantly increased plant height by 11.90% and 16.67%, respectively. Likes root length, plant height of PEG + MT + Hb treatment was also decreased significantly compared with PEG + MT treatment, and the percentage of decrease was 12.50% (Fig. 4b). These results indicated that CO and MT treatments can alleviate effectively drought stress. Meanwhile, the effect of CO and MT combined treatment on enhancing drought resistance of tomato seedlings was better than that of CO and MT treatment alone. The effect of MT on enhancing drought resistance of tomato seedlings was reduced by Hb, indicating that CO was involved in MT-enhanced drought resistance in tomato seedlings.

### Roles of exogenous MT and CO in the accumulations of Uro III, Proto IX, Mg-Proto IX and pchlide under drought stress

**Fig. 5:**
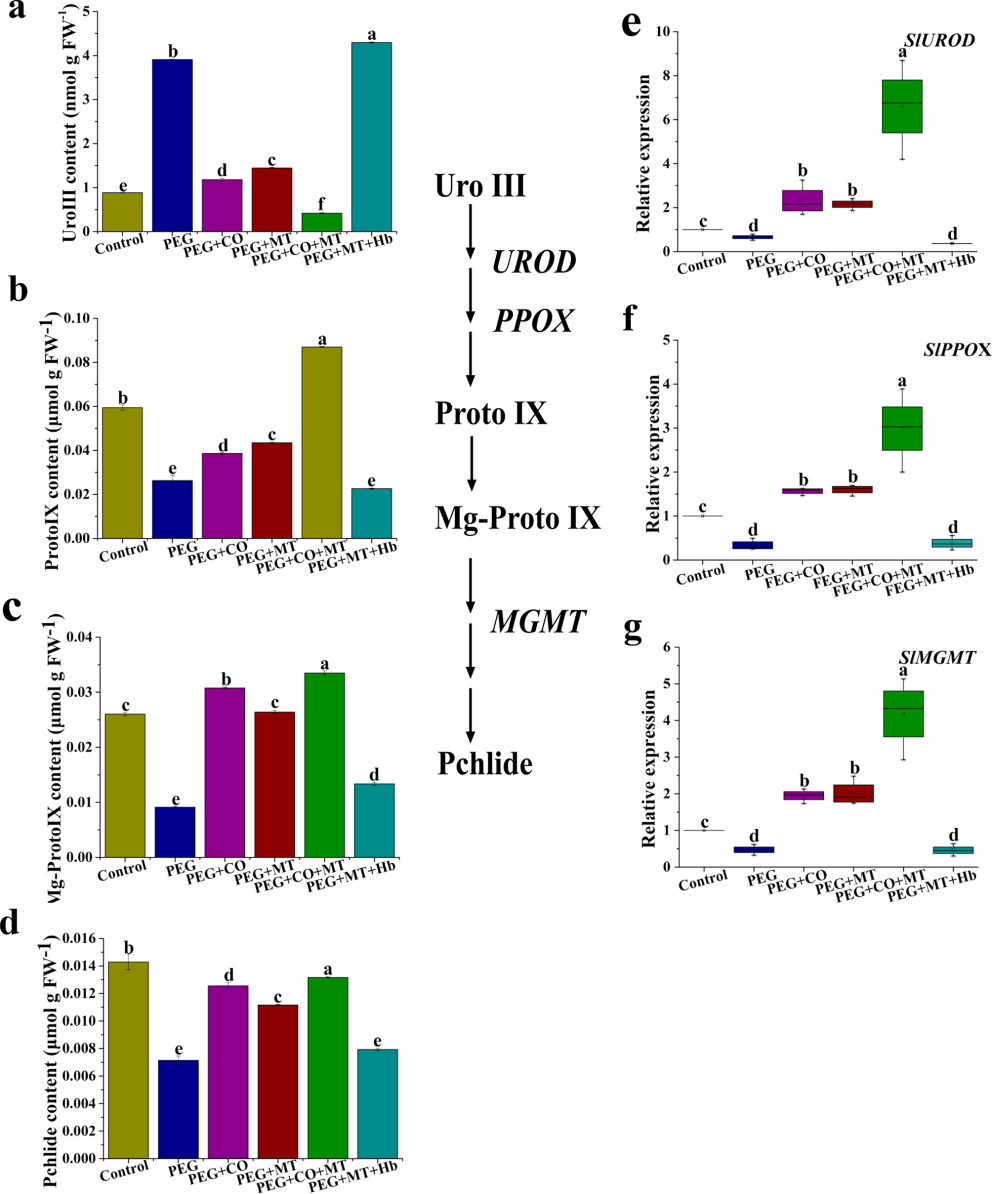
Effects of MT, CO, MT + CO, and MT + Hb on the levels of Uro III **(a)**, Proto IX **(b)**, Mg-Proto IX **(c)**, Pchlide **(d)** and the expression of *SlUROD***(e)**, *SlPPOX***(f)**, and *SlMGMT* (g) in tomato seedlings under drought stress. The data are the average of three replicates and are presented as means ± SE. Different letters indicate significant differences at *P* < 0.05 according to Duncan’s multiple range tests

As shown in Fig. 5a, the content of Uro III in PEG treatment significantly higher than that of the control. Compared with PEG treatment, a lower Uro III content was observed in PEG + MT or PEG + CO treatment. Uro III content in PEG + CO + MT treatment was markedly lower than PEG + MT or PEG + CO treatment. In PEG + MT + Hb treatment, Uro III content was significantly higher than that of PEG + MT treatment (Fig. 5a). As shown in Fig. 5b, c, d, the contents of Proto IX, Mg-Proto IX, and Pchlide were significantly decreased under drought stress compared with the control. Applications of CO and MT exhibited an obvious increase in Proto IX, Mg-Proto IX, and Pchlide under drought stress. PEG + CO + MT treatment had a significant higher Proto IX and Mg-Proto IX contents than PEG + CO or PEG + MT treatment. Compared with PEG + MT, PEG + MT + Hb significantly decreased Proto IX, Mg-Proto IX, and Pchlide contents (Fig. 5b, c, d). Moreover, the expression of *SlUROD, SlPPOX*, and *SlMGMT* were down-regulated significantly under drought stress compared with the control (Fig. 5e, f, g). CO or MT alone treatment up-regulated the expression of *SlUROD, SlPPOX*, and *SlMGMT* under drought stress. The expression levels of *SlUROD, SlPPOX*, and *SlMGMT* were obviously up-regulated by CO and MT co-treatment under drought stress compared with CO or MT alone treatment. Compared with PEG + MT treatment, the expression of *SlUROD, SlPPOX*, and *SlMGMT* were down-regulated in PEG + MT + Hb treatment (Fig. 5e, f, g).

### Roles of exogenous MT and CO in heme accumulation under drought stress

**Fig. 6:**
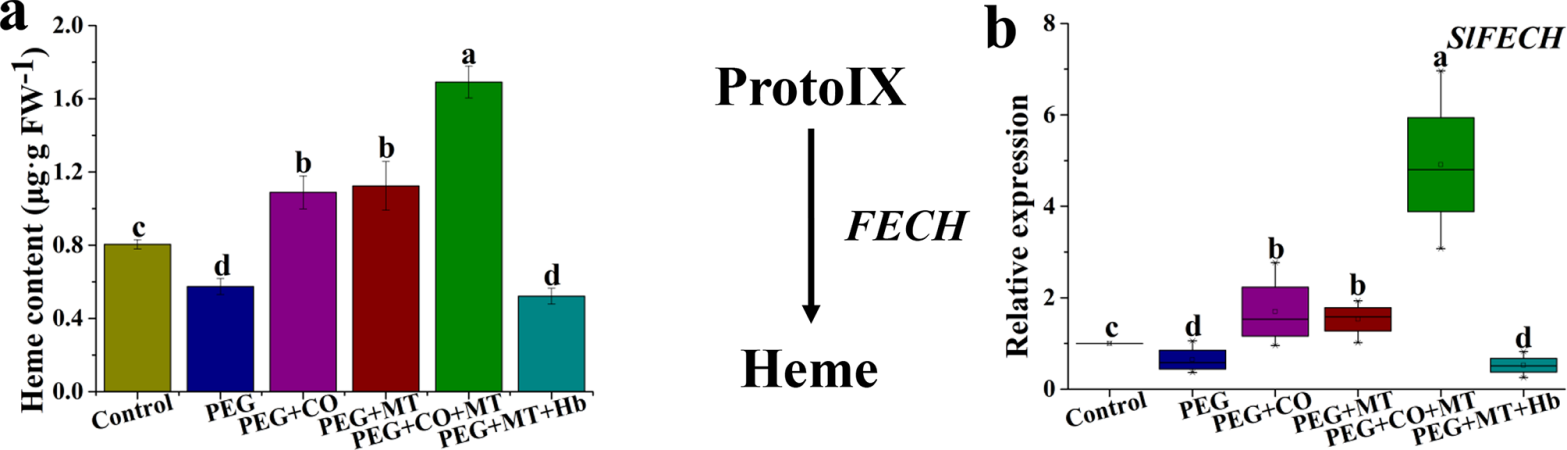
Effects of MT, CO, MT + CO, and MT + Hb on the level of heme **(a)** and the expression of *SlFECH***(b)** in tomato seedlings under drought stress. The data are the average of three replicates and are presented as means ± SE. Different letters indicate significant differences at *P* < 0.05 according to Duncan’s multiple range tests

As shown in Fig. 6a, PEG treatment significantly reduced heme content compared with the control. MT or CO treatment increased heme content under drought stress. Meanwhile, PEG + CO + MT treatment showed the highest heme content. PEG + MT + Hb treatment significantly reduced heme content compared with PEG + MT treatment (Fig. 6a). The results also revealed that the expression of *SlFECH* was down-regulated under drought stress (Fig. 6b). But applications of CO or MT up-regulate expression of *SlFECH* under drought stress. PEG + CO + MT treatment had the highest expression level of *SlFECH*. A significant down-regulation of *SlFECH* was observed in PEG + MT + Hb treatment compared with PEG + MT.

### Roles of exogenous MT and CO in Chl a and Chl b synthesis under drought stress

**Fig. 7:**
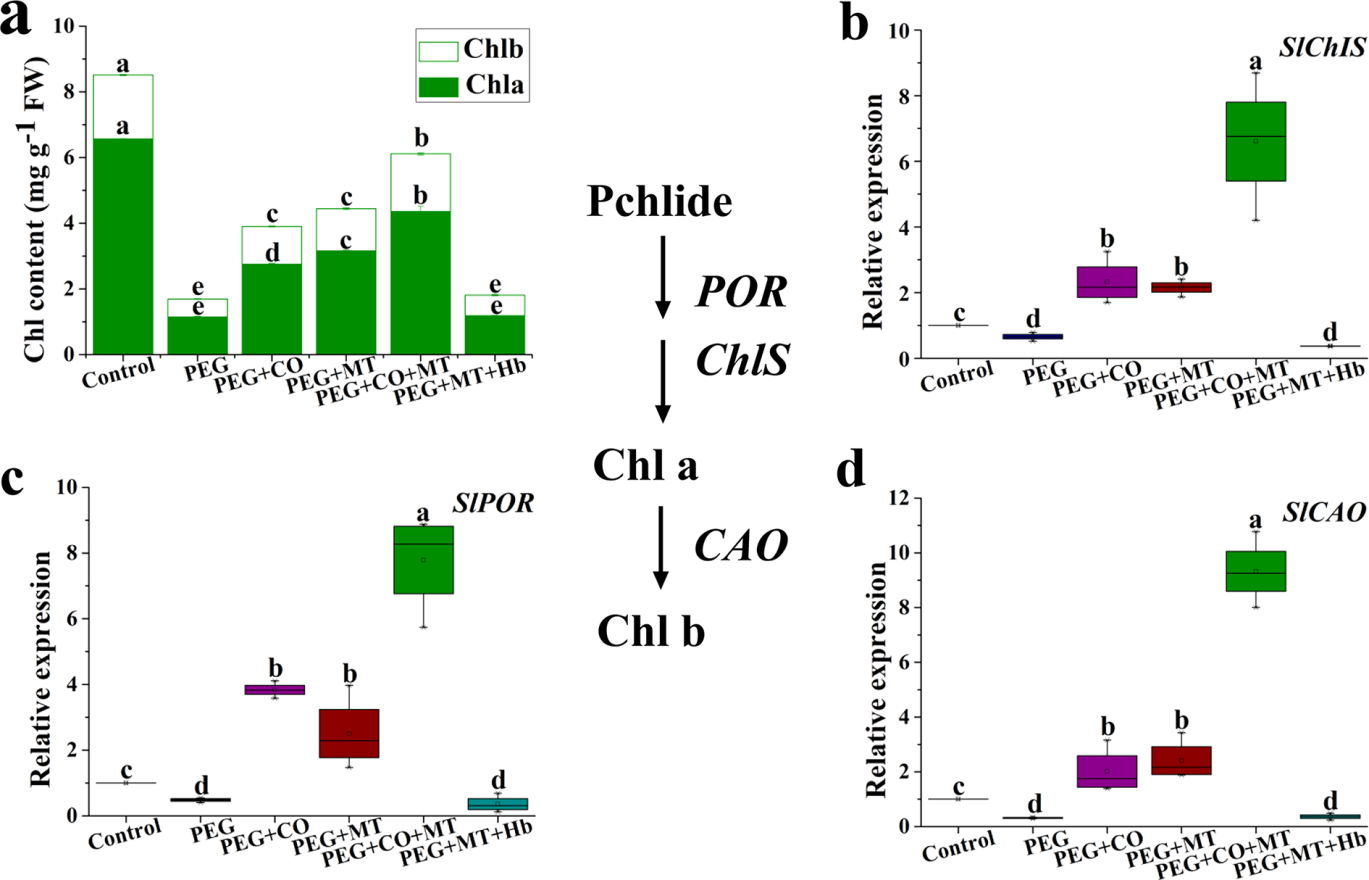
Effects of MT, CO, MT + CO, and MT + Hb on the levels of Chl a, Chl b, and total Chl **(a)** and the expression of *SlPOR***(b)**, *SlChlS***(c)**, and *SlCAO***(d)** in tomato seedlings under drought stress. The data are the average of three replicates and are presented as means ± SE. Different letters indicate significant differences at *P* < 0.05 according to Duncan’s multiple range tests

Drought stress significantly reduced the total chlorophyll content compared with the control (Fig. 7a). Exogenous CO or MT markedly increased chlorophyll content under drought stress. CO and MT co-treatment exhibited a higher chlorophyll content than CO or MT treatment alone. Compared with PEG + MT treatment, the chlorophyll content of PEG + MT + Hb treatment was significantly reduced (Fig. 7a). The key genes of chlorophyll synthesis *SlChlS*, *SlPOR*, and *SlCAO* were significantly down-regulated under drought stress compared with the control (Fig. 7b, c, d). Under drought stress, CO or MT treatment up-regulated expression of *SlChlS*, *SlPOR*, and *SlCAO*. A higher expression level of *SlChlS*, *SlPOR*, and *SlCAO* were recorded in PEG + CO + MT treatment compared with PEG + CO or PEG + MT treatment (Fig. 7b, c, d). PEG + MT + Hb treatment significantly down-regulated the expression of *SlChlS*, *SlPOR*, and *SlCAO* compared with PEG + MT treatment.

## Discussion

Drought influences severely plant growth and physiology, even plant cells [23]. When maize plants were subjected to drought stress, the leaf area, plant height, and grain yield were restrained markedly [54]. Growth, photosynthesis and nutrient uptake of apple plants under drought stress were reduced [23]. In our study, plant height and root length of tomato seedlings were significantly decreased by drought stress (Fig. 1). However, application of MT or CO significantly increased the root length and plant height under drought stress, and the effect of MT or CO on alleviating drought stress was concentration-dependent (Figs. 2 and 3). Under drought stress, 100 µM MT or 500 µM hemin application showed most significant effect on maintaining plant height and root length (Figs. 2 and 3). Meanwhile, the co-treatment of CO and MT was more effective to resist drought stress (Fig. 4). MT played a considerable role in cell protection against drought stress [23, 54]. MT can enhance drought tolerance by activating nitrogen assimilation-related enzymes such as nitrate reductase, nitrite reductase, glutamine synthetase, and glutamate synthase, and reducing H_2_O_2_ production in apple plants [23]. MT also regulated phytohormone homeostasis of drought-stressed maize seedlings such as elevating GA_3_, IAA and zeatin riboside levels, and decreasing ABA level [54]. CO, an important messenger molecule, has been shown to protect plant from drought and osmotic stress [35, 41]. The biosynthesis of CO in animal and plant was catalyzed by heme oxygenase-1 (HO-1) [33]. In the present study, hemin was used as CO donor to study the effect of CO on plant physiology, because it can up-regulate HO-1 to generate CO [30, 33, 41, 55, 56]. The adventitious root development and antioxidant enzymes activities were promoted by CO aqueous solution or hemin in cucumber under drought stress [41]. *COMT*-overexpression (*COMT*, a key gene of MT synthesis) *Arabidopsis* showed a higher survival rate than that of wild type under drought stress [57]. Endogenous heme oxygenase/CO system participated in the alleviation of drought-induced inhibition and oxidative damage in wheat seed germination [35]. In addition, Hb was thought to be a good CO scavenger to study the role of CO in plant [33, 41, 58, 59]. The application of Hb reversed the positive effect of CO on adventitious root development in cucumber under drought stress [41]. Han et al. [58] found that the role of CO in mediating cadmium-induced oxidative damage in alfalfa root was blocked by Hb. Our result showed that the effect of MT on alleviating drought stress was counteracted when CO was scavenged, indicating CO was an essential factor in MT-enhanced drought tolerance in tomato seedlings (Fig. 4).

Drought stress can cause ROS accumulation and restrain photosynthesis in plant [27]. Notably, the reduction of photosynthetic pigments could be a primary consequence of plant responding to drought stress [4]. We found that drought stress significantly affected chlorophyll and heme synthesis by increasing Uro III content, reducing accumulation of Proto IX, and down-regulating expression of *SlUROD* and *SlPPOX* (Fig. 5). Aarti et al. [60] reported that Proto IX and Pchlide accumulation were repressed in cucumber under oxidative stress. Moreover, under water stress, the accumulation of Pchlide and the activities of PPOX and POR were reduced in rice seedlings [53]. In the present study, heme and the expression of its synthesis-related gene *SlFECH* were down-regulated by drought stress (Fig. 6). Meanwhile, the intermediate products in chlorophyll branch including Mg-Proto IX and Pchlide were decreased significantly, and the expression of *SlMGMT* was also down-regulated under drought stress (Fig. 5). Furthermore, Chl a and Chl b contents in drought-stressed tomato seedlings were decreased significantly, similarly, the expression of *SlPOR*, *SlChlS*, and *SlCAO* involved in chlorophyll biosynthesis were down-regulated (Fig. 7). These studies indicated that plant that suffer from different environmental stresses may have similar variations in heme and chlorophyll biosynthesis.

**Fig. 8:**
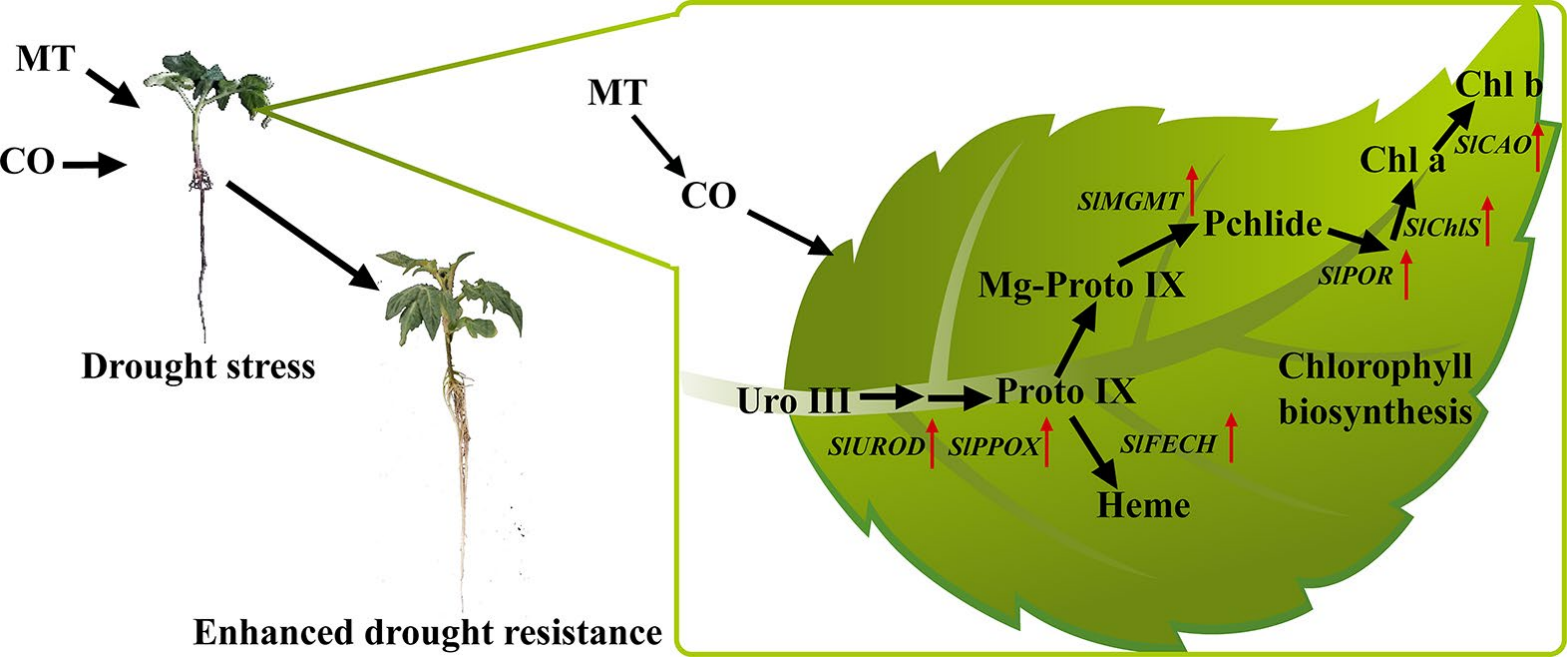
CO mediates MT-enhanced chlorophyll biosynthesis in tomato seedlings under drought stress. CO is involved in the MT-improved biosynthesis of chlorophyll and heme by increasing content of Proto IX, heme, Mg-Proto IX, Pchlide, Chl a and Chl b and up-regulating expression of *SlUROD, SlPPOX, SlFECH, SlMGMT, SlPOR, SlChlS*, and *SlCAO.* CO, carbon monoxide; MT, melatonin; Uro III, uroporphyrinogen III; Proto IX, protoporphyrin IX; Mg-Proto IX, Mg-protoporphyrin IX; Pchlide, potochlorophyllide; Chl a, chlorophyll a; Chl b, chlorophyll b

MT or CO treatment can promote the biosynthesis of chlorophyll and heme in drought-stressed tomato seedlings (Fig. 8). Application of MT and CO exhibited a prominent effect on increasing Proto IX, Mg-Proto IX, and Pchlide content, decreasing Uro III content, and up-regulating *SlUROD*, *SlPPOX* and *SlMGMT* expression (Fig. 5). Exogenous CO and MT also reversed the effects of drought stress on heme synthesis (Fig. 6), promoted Chl a and Chl b accumulation in drought-stressed seedlings (Fig. 7). Under drought stress, chlorophyll content was increased by MT in maize [54], coffee [26] and kiwifruit [4]. Thus, we concluded MT as a powerful and helpful molecule was involved in chlorophyll biosynthesis to alleviate drought stress in plant. In addition to MT, CO is also described as a promoter of chlorophyll accumulation in plant under stress. CO enhanced the Chl a, Chl b, and total chlorophyll concentrations of *Cassia obtusifolia* L. seedlings subjected to salinity stress [59]. chlorophyll content of Cu-stressed *Chlamydomonas reinhardtii* was increased by CO treatment [61]. CO alleviated chlorophyll reduction in *Arabidopsis* under iron-deficiency condition [40]. In the study of Chen et al. [41], CO was strongly involved in H_2_-induced chlorophyll accumulation in cucumber under drought stress. In this study, we demonstrated that CO can elevate chlorophyll content in drought-stressed tomato seedlings. Therefore, CO functions as an important regulatory molecule involving in chlorophyll biosynthesis and stress-resistant in plant. Interestingly, under drought stress, the co-treatment of MT and CO exhibited the more significant effect on promoting heme and chlorophyll biosynthesis than that of MT or CO treatment alone. Additionally, Hb application counteracted the influence of MT on chlorophyll and heme synthesis, suggesting that CO was a vital factor in MT-mediated chlorophyll biosynthesis. However, the potential mechanism of CO interacts with MT in plant is still lack of understanding. In the animal experiments, CO can stimulate pineal cells to release MT [62]. Interestingly, MT also up-regulated HO-1 activity in rats [63]. Further investigations should be established to characterize the molecular mechanism in plant. It is worth noting that one signal molecule may play a key role in the relationship between CO and MT, and that is nitric oxide (NO). The up-regulation of *HO-1*, a gene related to CO biosynthesis, induced NO pathway in wheat under osmotic stress, suggesting that NO was involved in the enhancement of osmotic resistance [35]. CO improved the resistance of *Baccaurea ramiflora* to low temperature via NO-induced glutathione homeostasis [45]. On the other side, NO was involved in MT-enhanced cadmium resistance of tomato seedlings [18]. NO also functioned as a downstream signal of MT improving plant resistance to cold and alkaline stress [48, 64].

## Conclusion

Exogenous MT and CO can alleviate the lower plant height and root length under drought stress and facilitate the biosynthesis of chlorophyll and heme by up-regulating *SlUROD*, *SlPPOX*, *SlMGMT*, *SlFECH*, *SlPOR*, *SlChlS*, and *SlCAO* expression in tomato. The combined treatment of MT and CO expressed a more significant effects on drought resistance compared with MT or CO treated alone. The addition Hb blocked the roles of MT in chlorophyll biosynthesis, indicating that CO could be a downstream signal molecule of MT-enhanced drought resistance by promoting chlorophyll biosynthesis. Therefore, we suggested that this positive role of MT and CO offered new insight into the field of drought stress, and this finding may provide theoretical basis in the drought resistance of plant.

## Materials and methods

### Plant material and growth conditions

The tomato ‘Micro-Tom’ (*Solanum lycopersicum* L.) was used as experimental material in the current study. The healthy tomato seeds were selected and disinfected with 1% of NaClO solution. Subsequently, seeds were immersed in pure water and placed in constant temperature shaker (180 rpm, 28℃). The germinated seeds were planted in the soil mixture containing turfy soil and perlite 3:1 (v/v) for 2 weeks. Then the tomato seedlings with 4 leaves were transferred to Hoagland solution and incubated at 25 ± 1 ℃ for 16 h light (250 µ mol m^− 2^ s^− 1^ photons irradiance) and 20 ± 1 ℃ for 8 h dark. The seedlings of uniform growth were collected with different concentrations another 1 week: firstly, PEG (1%, 5%, and 10%, m/v); secondly, PEG (5%) + MT (10, 50, 100, and 200 µM) and PEG (5%) + Hemin (a donor of CO, 100, 500, 1000, and 2000 µM); finally, PEG (5%) + MT (100 µM), PEG (5%) + Hemin (500 µM), PEG (5%) + MT (100 µM) + Hemin (500 µM), and PEG(5%) + MT (100 µM) + Hb (a CO scavenger, 1 g L^− 1^). Seedlings treated with the Hoagland solution served as the control.

### Plant height and root length

Plant height and root length were counted manually by using the vernier caliper. Plant height was measured from the basal part of stem to the terminal of bud of the main stem. Root length was defined as the portion from the base of the stem to the end of the longest root [23].

### Uro III content

Uroporphyrinogen III (Uro III) was measured in the leaves according to the method described by Bogorad [65] with some modifications. Firstly, 1 g of fresh leaf was ground with 5 mL Tris-HCl buffer (pH 7.2) in ice bath, and then the homogenate was centrifuged at 5000 *g* for 15 min at 4 ℃. After that, the pH of supernatant was modulated at 4.0 by adding glacial acetic acid, and centrifuged at 5000 *g*. Subsequently, the sediment was immersed in distilled water and centrifuged at 5000 *g* for 15 min at 4 ℃. Precooled 4 mL ammonia spirit was used to extract Uro III from the sediment twice, then centrifuged at 5000 *g*. Then the supernatant was evaporated to dryness at 55℃. The sediment was mixed with 4 mL sulfuric acid-methanol containing 5% sulfuric acid, 95% methanol to esterify for 48 h. After that, 20 mL distilled water was added in the mixture, the pH of homogenate was maintained at 4.0 by saturated sodium acetate, and 4 mL chloroform was used to extracted the mixture. Then homogenate was evaporated to dryness at 55 ℃, sediment was dissolved in 4 mL chloroform. Finally, the absorbance of supernatant was measured at 405.5 nm, and the calculation of Uro III content was performed by the following formula, Uro III (nmol g FW^− 1^) = [A405 / (ε × d)] × V/FW × 10^9^. In the formula, ε = 5.48 × 10^5^ L mol^− 1^ cm^− 1^ is the molar extinction coefficient of Uro III under 405.5 nm; d = 1 cm is the optical path length of determine solution; V = 0.004 L is the dissolved volume of Uro III; FW = 1 g is the weight of fresh sample; The 10^9^ is to convert the unit from mol g FW^− 1^ to nmol g FW^− 1^.

### Proto IX, Mg-Proto IX and pchlide contents

The measurements of protoporphyrin IX (Proto IX), Mg-protoporphyrin IX (Mg-Proto IX), and protochlorophyllide (Pchlide) were performed according to Hodgins and Van Huystee [66] with minor modifications. Fresh leaf (0.3 g) was ground and homogenized with 25 mL 80% alkaline acetone, then stored in dark condition until the tissue was bleached. After that, the absorbances of supernatant were measured at 575 nm, 590 nm, and 628 nm. The calculation was performed according to the formulas reported by Shen et al. [67]. In the formulas, *V* is the dissolved volume of determined solution; *FW* is the weight of fresh sample.

Proto IX (µmol g FW^− 1^) = (0.18016 × A575 − 0.04036 × A628 − 0.04515 × A590) × V/FW.

Mg - Proto IX (µmol g FW^− 1^) = (0.06077 × A590 − 0.01937 × A575 − 0.003423 × A628) × V/FW.

Pchlide (µmol g FW^− 1^) = (0.03563 × A628 + 0.007225 × A590 − 0.02955 × A575) × V/FW.

### Heme content

Heme was measured according to the methods of Marsh et al. [68] with some modifications. Firstly, 2 g of fresh leaf was ground in liquid nitrogen, and then mixed with 5 mL of extract I (10% 0.1 M ammonia and 90% acetone). Then, the mixture was centrifuged at 8000 *g* for 10 min. Repeated this process until the chlorophyll was completely removed. Subsequently, 5 mL of extract II (80% acetone, 16% dimethyl sulfoxide, and 4% concentrated sulfuric acid) was added into the sediment, and centrifuged at 8000 *g* for 10 min. Then 3 mL diethyl ether, 2 mL saturated sodium chloride, and 10 mL deionized water were mixed with the sediment, and centrifuged at 1000 *g* for 1 min. Finally, the supernatant was added into 0.7 mL ethanol, and the absorbance was determined at 386 nm. The heme concentration was calculated with a standard curve of heme reference standards. The concentrations of heme standard curve were 0, 1, 3, 5, 7, and 10 µg mL^− 1^.

### Chlorophyll content

The chlorophyll of tomato leaves was extracted as described by Porra et al. [69]. Fresh leaf (0.2 g) was homogenized with 15 mL 80% buffered aqueous acetone until the leaf was completely bleached, then filled up to 25 mL by adding 80% acetone. Chlorophyll was quantified by measuring the absorbances of supernatant at 646 and 663 nm, and the content of chlorophyll (Chl a and Chl b) was calculated using the following formulas according to Lichtenthaler [70].

Chl a (mg g^− 1^ FW) = (12.21 × A663 − 2.81 × A646) × V/FW.

Chl b (mg g^− 1^ FW) = (20.13 × A646 − 5.03 × A663) × V/FW.

### Real‑time RT‑PCR analysis

For quantitative real-time PCR (qRT-PCR) analyses, total RNA was isolated from 0.5 g tomato leaf samples by using Trizol reagent (Invitrogen, Gaithersburg, MD) according to the method of Lu et al. [71]. Then the extracted RNA (500 ng) from different treatment was reverse-transcribed to synthesize cDNA in a 10 µL reaction containing 2 µL of AMV reverse transcriptase XL (AG, China) and 2.5 µM random primer. ABI Step One Plus system (Applied Biosystems, Carlsbad, CA) and SYBR® Premix Ex Taq™ II (AG, China) were used to cDNA synthesis according to the manufacturer’s instructions. The PCR cycling program was run at 95 ℃ for 15 min firstly, monitored for 40 cycles at 95 °C for 10s, and then annealing at 60 ℃ for 20 s. The primers used for PCR analysis were listed in Table 1, and *actin* was used as the reference gene. Afterward, the transcript levels of each gene were calculated as described by Livak and Schmittgen [72].

**Table 1:** Primers used for gene expression analysis

Gene name	Accession number	Forward primer sequence	Reverse primer sequence
*SlCAO*	LOC101261422	GCAGCCTAGAAGATCCCTCAATGTG	ATCAGCGGAGAAAGCAACAGGATAC
*SlChlS*	LOC101246752	TGTTATAGGCAGGGCATGACTTTCC	TGGTGGAGCTGAGTAGATGTAGGAG
*SlFECH*	LOC101248153	ATGCCTGCTTGCCATTCCTCAC	GACAATCCTCCACCTACACTGCTTC
*SlMGMT*	LOC101267302	TCCGCCGCTACCGACATCTC	CCGACCTCCTCCGCCTGAAG
*SlPOR*	LOC101244717	TGGACCTCGCCTCTCTTGACAG	AAACAGCAGCATTAGCAACCAACAC
*SlPPOX*	LOC101259974	CGCCGTCCTCCTCCTCATCC	ACCGTGTTCTCCAGCCATTGC
*SlUROD*	LOC101260578	AGAAAGTGCCCACAAACACCTCTTG	GCCGCCTTCCATCTGCCATATC
*Actin*	NC_015447	AATGAACTTCGTGTGGCTCCAGAG	ATGGCAGGGGTGTTGAAGGTTTC

### Statistical analysis

The experimental data in this study were expressed as the means ± standard error (SE) of at least three replicates, with each replicate consisting of 30 tomato seedlings. The results were analyzed by using Microsoft Excel 2019 (Microsoft Inc., Redmond, WA, USA) and SPSS 26.0 (IBM SPPS Inc., Chicago, IL, USA). And the figures in this paper were prepared with OriginPro 2022 (OriginLab Institute Inc., Northampton, MA, USA). For statistical analysis, the significance of the differences among the mean values of each treatment was determined according to Duncan’s multiple range test (*P* < 0.05).

## Data Availability

The datasets used and analysed during the current study are available from the corresponding author on reasonable request.
